# Phenotypic and genotypic characterization of locally isolated *Salmonella* strains used in preparation of *Salmonella* antigens in Egypt

**DOI:** 10.14202/vetworld.2016.1435-1439

**Published:** 2016-12-17

**Authors:** Hazem Mohammed Ibrahim, Dalia Ahmed Mohammed Abd El-Moaty, Hanan Ali Ahmed, Mona Ibrahim El-Enbaawy

**Affiliations:** 1 Department of Bacterial Sera and Antigens Research, Veterinary Serum and Vaccine Research Institute, Abbasia, Cairo, Egypt; 2 Genetic Engineering Research Department, Veterinary Serum and Vaccine Research Institute, Abbasia, Cairo, Egypt; 3 Central Laboratory for Evaluation of Veterinary Biologics, Cairo, Egypt; 4 Department of Microbiology, Faculty of Veterinary Medicine, Cairo University, Cairo, Egypt

**Keywords:** characterization, duplex polymerase chain reaction, multiplex polymerase chain reaction, *Salmonella* spp

## Abstract

**Aim:**

This work was conducted to study the phenotypic and genotypic characterization of locally isolated *Salmonella* strains (*Salmonella* Pullorum, *Salmonella* Enteritidis, and *Salmonella* Typhimurium) from poultry used in the preparation of *Salmonella* antigens in Egypt.

**Materials and Methods:**

The phenotypic characterization of *Salmonella* strains was done using standard microbiological, biochemical, and serological techniques. Molecular identification was done using different sets of primers on different genes using different polymerase chain reaction (PCR) techniques.

**Results:**

The phenotypic characterization of *Salmonella* strains was confirmed. Molecular identification revealed detection of 284 bp fragment of InvA gene in all studied *Salmonella* strains. Furthermore, multiplex PCR was used for more confirmation of being *Salmonella* spp., generally at 429 bp as well as genotyping of *Salmonella* Typhimurium and *Salmonella* Enteritidis at 559 and 312 bp, respectively, in one reaction.

**Conclusion:**

The locally isolated field *Salmonella* strains were confirmed phenotypically and genotypically to be *Salmonella* Enteritidis, and *Salmonella* Typhimurium and could be used for the preparation of *Salmonella* antigens.

## Introduction

*Salmonella* organisms are responsible for a variety of acute and chronic diseases in poultry, animals, and humans [1]. *Salmonella* bacteria are facultative intracellular pathogens causing localized or systemic infections, in addition to a chronic asymptomatic carrier state. Many different serotypes of *Salmonella* have been isolated from poultry, most of them have a public health significance, but some include *Salmonella* Typhimurium, *Salmonella* Enteritidis, *Salmonella* Pullorum, and *Salmonella* Gallinarum can cause considerable losses in birds of less than a few weeks of age [2]. *Salmonella* enterica serovars Typhimurium and *Salmonella* enterica serovars Enteritidis are the most frequently isolated serovar from foodborne outbreaks throughout the world [3]. *Salmonella* Pullorum is a typical bacterial disease that has threaten the modern poultry industry over the past years. Chicken becomes the carrier in the spread of *Salmonella* Pullorum and may cause economic losses worldwide through mortality, morbidity, and reductions in egg production [4]. Establishing conventional methods was applied to detect and identify *Salmonella* include selective enrichment and plating followed by biochemical tests and serological identification [5]. In general, these techniques are time-consuming since they give only presumptive results after 3-4 days and definitive results after 5-6 days [6]. However, because of controversy in interpreting results, low sensitivity and specificity of these methods, rapid detection methods, such as DNA or RNA probing, immuno-detection methods and nucleic acid hybridization have been developed, but they do not have enough sensitivity and specificity [7].

*In vitro* amplification of DNA by the polymerase chain reaction (PCR) method is a powerful tool in microbiological diagnostics [8]. Several genes have been used to detect *Salmonella* in natural environmental samples as well as food and fecal samples. Virulence chromosomal genes - including invA, invE and himA, phoP - are target genes for PCR amplification of *Salmonella* species [9]. The invA gene of *Salmonella* contains sequences unique to this genus and has been proved as a suitable PCR target with potential diagnostic applications [10]. Multiplex PCR simultaneously detecting several pathogens in a single-tube reaction and has the potential of saving time and effort, lowering testing-related laboratory cost [11]. Typing of *Salmonella* Enteritidis and Typhimurium using multiplex PCR reaction is depending on sefA gene which encodes for SEF14 fimbrial antigen characteristic for *Salmonella* Enteritidis while *fliC* gene variable region encoding for flagellin H1 was characteristic for *Salmonella* Typhimurium [12].

The objective of this work was to characterize the locally isolated *Salmonella* strains used in the preparation of *Salmonella* antigens in Egypt by both phenotypic and genotypic methods.

## Materials and Methods

### Ethical approval

The approval from the Institutional Animal Ethics Committee to carry out this study was not required as no invasive technique was used.

### Bacterial strains

Three local field *Salmonella* strains (*Salmonella* Pullorum, *Salmonella* Enteritidis, and *Salmonella* Typhimurium) isolated from chickens, kindly obtained from Bacterial Sera and Antigens Research Department, Veterinary Serum and Vaccine Research Institute, Abbasia, Egypt were used to study their phenotypic and genotypic characterization. All isolates were confirmed as *Salmonella* different types using both morphological and biochemical identification [13]. Serological typing was performed using reference *Salmonella* antisera [14].

### Total DNA extraction of Salmonella isolates

That was performed by boiling the overnight incubated culture broth for 10 min in dry bath and centrifuged at 5000 ×*g* for 10 min. The supernatant was used for amplification by PCR using *Salmonella*-specific primers. The extract was divided into aliquots and kept at −20°C until use as PCR template [15].

### Primers set

Primers used were supplied by Metabion (Germany) and summarized in Table-1. For diagnosis of *Salmonella* spp. generally, a primer set was used for amplification of 284 bp of InvA gene [10]. Another primer sets were used for general identification of *Salmonella* spp. as well as typing of *Salmonella* Typhimurium and *Salmonella* Enteritidis in a multiplex PCR reaction [12]. Typing of *Salmonella* Pullorum was done using a duplex PCR, to differentiate between *Salmonella* Gallinarum and *Salmonella* Pullorum depending on the presence of speC gene in both strains but glgC gene is unique for *Salmonella* Gallinarum only [16].

**Table-1:** Primer sets for *Salmonella* strains PCR.

Primer set	*Salmonella* strain	Target gene	Primer sequence 5’ 3’	Length	Amplicon fragment (bp)
S139	*Salmonella* spp.	*invA* gene	GTG AAA TTA TCG CCA CGT TCG GGC AA	26	284
S141			TCA TCG CAC CGT CAA AGG AAC C	22	
ST11	*Salmonella* spp.	Randomly cloned chromosomal fragment	AGCCAACCATTGCTAAATTGGCGCA	25	429
ST15			GGTAGAAATTCCCAGCGGGTACTG	24	
Fli 15	*Salmonella* Typhimurium	*fliC*	CGG TGT TGC CCA GGT TGG TAA T	22	559
Tym			ACT CTT GCT GGC GGT GCG ACT T	22	
Sef167	*Salmonella* Enteritidis	*SefA* gene	AGG TTC AGG CAG CGG TTA CT	20	312
Sef478			GGG ACA TTT AGC GTT TCT TG	20	
SG-L	*Salmonella* Pullorum	*glgC*	GAT CTG CTG CCA GCT CAA	18	252
SG-R			GCG CCC TTT TCA AAA CAT A	19	
SGP-L		*speC*	CGG TGT ACT GCC CGC TAT	18	174
SGP-R			CTG GGC ATT GAC GCA AA	17	

PCR=Polymerase chain reaction

### PCR amplification

Amplification was performed as following: 12.5 µl of ×2 Dream Taq Green PCR Master Mix (Fermentas), 100 pmol of upstream primer, 100 pmol of downstream primer, 4 µl of template DNA and nuclease-free water up to 25 µl using thermal cycler PerkinElmer Gene Amp PCR system 9700. Amplification conditions of 284 bp of InvA gene where the thermal cycler were adjusted to 1 cycle at 95°C for 1 min, then 35 cycles at 95°C for 1 min, 64°C for 30 s, 72°C for 30 s followed by 1 cycle at 94°C for 4 min [17]. For multiplex PCR, the amplification conditions were adjusted to 1 cycle at 94°C for 1 min, 35 cycles at 94°C for 30 s, 56°C for 1 min 30 s, 72°C for 30 s followed by 1 cycle at 72°C for 10 min [12]. Duplex PCR was performed [16,18], with a wide range of annealing temperatures, where PCR conditions were 1 cycle at 95°C for 5 min, 35 cycle of 95°C for 30 s, 55-65°C for 30 s, and 72°C for 30 s followed by a final extension step at 72°C for 10 min. Sterile DNase and RNase free water were used as negative PCR control.

### Analysis of PCR products

All amplified products were analyzed by electrophoresis using 1-1.5% agarose gel (Applichem, Germany, GmbH) and visualized by ultraviolet transilluminator after gel staining with ethidium bromide stain (Fisher). The product size was measured using 100 bp DNA Ladder (Fermentas) that was used as a marker for PCR products. The gel was photographed by a gel documentation system (Alpha Innotech, Biometra), and the data were analyzed through computer software.

### DNA sequence and analysis

PCR fragment of speC gene of *Salmonella* Pullorum was purified with agarose gel extraction kit Qiaquick (Qiagen, Germany). Sequence analysis of this fragment was performed using the same PCR primers (Macrogen Inc., Seoul, Korea).

## Results

**Figure-1:**
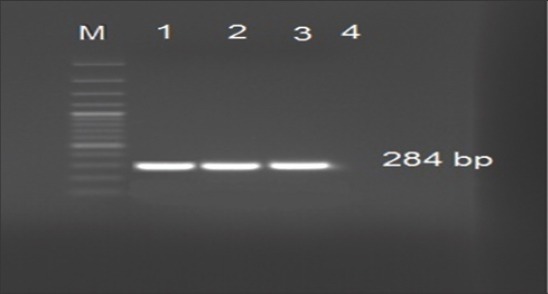
Agarose gel electrophoresis showing amplification of 284 bp of Inv A gene of *Salmonella* spp. Lane M: 100 bp DNA ladder (Fermentas), Lane 1: *Salmonella* Typhimurium, Lane 2: *Salmonella* Enteritidis, Lane 3: *Salmonella* Pullorum, Lane 4: Negative polymerase chain reaction control.

In this study, three locally fields isolated *Salmonella* strains used in preparation of *Salmonella* antigens in Egypt were tested and confirmed to be *Salmonella* species phenotypically by culturing and biochemical testing. Furthermore, these strains were confirmed serologically to be *Salmonella* Pullorum, *Salmonella* Enteritidis, and *Salmonella* Typhimurium (Table-2). On the other hand, strains were confirmed to be related to *Salmonella* spp. by invA specific PCR methods as all isolates showed positive bands at 284 bp (Figure-1).

**Table-2:** Results of serotyping of *Salmonella* strains.

*Salmonella* groups and types	Antigenic formula		

	O	H	

		1	2
*Salmonella* Pullorum	1, 9, 12		
*Salmonella* Typhimurium	1, 4, 5, 12	I	1, 2
*Salmonella* Enteritidis	1, 9, 12	g, m	(1, 7)

( )=May be absent

**Figure-2:**
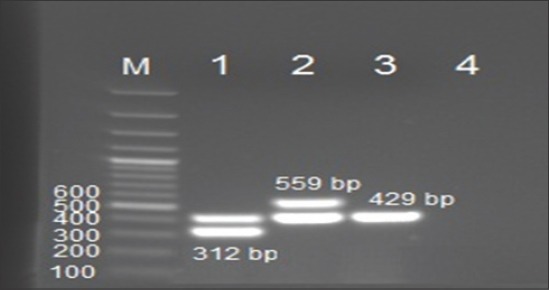
Genotyping of *Salmonella* strains by multiplex polymerase chain reaction (PCR). Lane M: 100 bp DNA ladder (Fermentas), All strains shared the same band at 429 bp which is general for all *Salmonella* spp. Lane 1 showed band at 312 bp specific for *Salmonella* Enteritidis. Lane 2 showed band at 559 bp specific for *Salmonella* Typhimurium, Lane 3 *Salmonella* Pullorum was negative for both *Salmonella* Typhimurium and *Salmonella* Enteritidis and showed only band 429 bp general for all *Salmonella* spp., Lane 4: Negative PCR control.

Genotype identification was done using multiplex PCR assay with simultaneous characterization of *Salmonella* spp. generally. The results obtained showed that the three used strains were positive by PCR primers set (ST11, ST15) showing specific bands at 429 bp (Figure-2) for all *Salmonella* spp. Multiplex PCR could differentiate between *Salmonella* Enteritidis and *Salmonella* Typhimurium that showing sharp specific bands at 312 and 559 bp, respectively (Figure-2).

**Figure-3:**
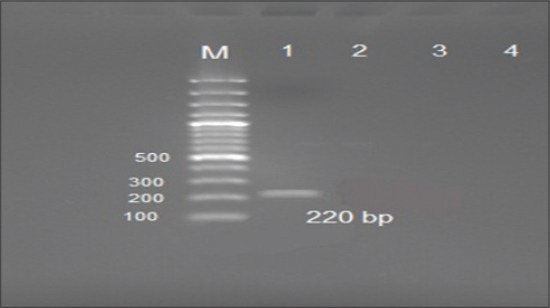
Duplex polymerase chain reaction (PCR) analysis to differentiate between biovars Gallinarum and Pullorum, Lane M: 100 bp DNA ladder (Fermentas), Lane 1: *Salmonella* Pullorum showing band at 220 bp, Lane 2: *Salmonella* Typhimurium, Lane 3: *Salmonella* Enteritidis, Lane 4: Negative PCR control.

Duplex PCR for identification of *Salmonella* Pullorum results as shown in Figure-3 revealed a band at 220 bp at 60°C annealing temperature only. None other bands were obtained by repeating the test with different PCR conditions. Furthermore, this test showed negative results or no product when tested with *Salmonella* Typhimurium and *Salmonella* Enteritidis. This band was purified for sequence analysis. The data obtained from sequence analysis of this fragment (data not shown) showed that this PCR fragment is not related to speC or glgC genes at all but it was related to yejBEF gene which is common among many other *Salmonella* spp.

## Discussion

*Salmonella* contamination of eggs has been identified as a public health concern worldwide. Globally, *Salmonella* is one of the most prevalent causes of foodborne illness [19]. Culture techniques are universally recognized as standard methods for detection of bacterial pathogens, such as *Salmonella* in foodstuffs [20]. These techniques generally take longer time [8] and are less sensitive compared to PCR-based methods [21]. InvA gene specific PCR method is the most used in diagnostic and research laboratories, and *Salmonella* identification by molecular techniques is the simplest and less expensive method [10].

In this study, all the three locally field isolated strains used in the preparation of *Salmonella* antigens in Egypt were tested and confirmed phenotypically to be *Salmonella* Pullorum, *Salmonella* Enteritidis and *Salmonella* Typhimurium by culturing, biochemical testing and serological characterization. These strains were confirmed to be related to *Salmonella* spp. by invA specific PCR methods as all isolates showed positive bands at 284 bp (Figure-1) which agree with the previously reported results [10,17]. Genotype identification was done using multiplex PCR assay with simultaneous characterization of *Salmonella* spp. generally. The results obtained showed that the three used strains were positive by PCR primers set (ST11, ST15) showing specific bands at 429 bp (Figure-2) for all *Salmonella* spp. Multiplex PCR could differentiate between *Salmonella* Enteritidis and *Salmonella* Typhimurium that showing sharp specific bands at 312 and 559 bp, respectively (Figure-2). Using the conventional PCR technique is a convenient tool for rapid and accurate identity of different *Salmonella* spp. as well as genotypic characterization of different *Salmonella* type either *Salmonella* Typhimurium, *Salmonella* Enteritidis and can be used as confirmatory tool with high sensitivity even if biochemical or serological tests were not available or the time factor is critical [6,12].

Duplex PCR results as shown in Figure-3 revealed a band at 220 bp. It was not the expected specific size of *Salmonella* Pullorum at 174 bp [16] but it was the only band obtained at different PCR conditions as well as it was negative with other used strains. The possibility of insertions was reported previously through mapped genomes of serovar Pullorum [22] and its comparisons with the genomes of other *Salmonella* serovars revealed several insertions, deletions, and rearrangements in serovars Gallinarum [23]. All these results drove us to suspect that the resulted band at 220 bp was due to nucleotide insertion either characteristic for Egyptian field isolates or due to serial passage. For clarification of this conflict, this band was purified for sequence analysis. The data obtained from sequence analysis of this fragment (data not shown) showed that this PCR fragment is not related to speC or glgC genes at all but it was related to yejBEF gene which is common among many other *Salmonella* spp. [24] that contributes to the virulence and antimicrobial resistance [25]. The false positive band at nonspecific size 220 bp may be due to false priming of downstream primer of speC gene at yejBEF gene within *Salmonella* genome as the primer design was based on an 11 bp deletion in the *glgC* gene and a 4 bp deletion in *speC*. Furthermore, it was found that glgC gene was a pseudogene in *Salmonella* Gallinarum while speC was a pseudogene in both biovars. In bacterial genomes, pseudogenes are continually created from ongoing mutational processes and are subject to degradation and removal by further accumulation of mutations. Their retention time seems to be extremely short and, even in very closely related bacteria, they tend to be deleted at a relatively rapid rate [26]. All these findings decreased the sensitivity and reliability of this duplex PCR. More investigations are required for rapid and easy identification of *Salmonella* Pullorum, as the previously reported conventional DNA-based methods are not feasible due to a high level of sequence similarities among *Salmonella* serovars as well as the limitation in resolution between biovars Gallinarum and Pullorum [27,28]. Furthermore, post-PCR steps as RFLP is laborious and time-consuming [29,30].

## Conclusion

The locally isolated field *Salmonella* strains were confirmed phenotypically and genotypically to be *Salmonella* Enteritidis and *Salmonella* Typhimurium and could be used for the preparation of *Salmonella* antigens. Further studies are required to develop and establish rapid and accurate protocols for genotyping of *Salmonella Pullorum*.

## Authors’ Contributions

HMI and HAA designed the work. HMI, DAMA and HAA conducted the research work. Data analysis and manuscript were written by HMI, DAMA and HAA under the guidance of MIE. All the authors have read and approved the final manuscript.
